# Characterization of rat glutathione transferases in olfactory epithelium and mucus

**DOI:** 10.1371/journal.pone.0220259

**Published:** 2019-07-24

**Authors:** Jean-Marie Heydel, Franck Menetrier, Christine Belloir, Francis Canon, Philippe Faure, Frederic Lirussi, Evelyne Chavanne, Jean-Michel Saliou, Yves Artur, Marie-Chantal Canivenc-Lavier, Loïc Briand, Fabrice Neiers

**Affiliations:** 1 University Bourgogne Franche-Comté, Faculty of Health Sciences, Dijon, France; 2 CSGA, Laboratory of taste and olfaction: from the molecule to behavior, University Bourgogne Franche-Comté, INRA, CNRS, France; 3 Université de Bourgogne, Centre Hospitalier Universitaire de Dijon, INSERM, U1231, Lipides Nutrition Cancer, Équipe labellisée Ligue Nationale contre le Cancer, Dijon, France; 4 University of Lille, CNRS, INSERM, CHU Lille, Pasteur Institute of Lille, U1019-UMR8204-CIIL-Center for Infection and Immunity of Lille, Lille, France; Universite Paris Diderot, FRANCE

## Abstract

The olfactory epithelium is continuously exposed to exogenous chemicals, including odorants. During the past decade, the enzymes surrounding the olfactory receptors have been shown to make an important contribution to the process of olfaction. Mammalian xenobiotic metabolizing enzymes, such as cytochrome P450, esterases and glutathione transferases (GSTs), have been shown to participate in odorant clearance from the olfactory receptor environment, consequently contributing to the maintenance of sensitivity toward odorants. GSTs have previously been shown to be involved in numerous physiological processes, including detoxification, steroid hormone biosynthesis, and amino acid catabolism. These enzymes ensure either the capture or the glutathione conjugation of a large number of ligands. Using a multi-technique approach (proteomic, immunocytochemistry and activity assays), our results indicate that GSTs play an important role in the rat olfactory process. First, proteomic analysis demonstrated the presence of different putative odorant metabolizing enzymes, including different GSTs, in the rat nasal mucus. Second, GST expression was investigated *in situ* in rat olfactory tissues using immunohistochemical methods. Third, the activity of the main GST (GSTM2) odorant was studied with *in vitro* experiments. Recombinant GSTM2 was used to screen a set of odorants and characterize the nature of its interaction with the odorants. Our results support a significant role of GSTs in the modulation of odorant availability for receptors in the peripheral olfactory process.

## Introduction

Odorant molecules are perceived by mammals upon their initial binding to the olfactory receptors localized in the cilia of the sensory neurons of the olfactory epithelium (OE). To reach the olfactory receptors, odorants cross through an aqueous layer of mucus lining the epithelium. The proteins contained in the olfactory mucus have been previously characterized in humans and mice [1,2]. These proteins, which are involved in many functions, such as antimicrobial resistance and protein folding, also include odorant transporters and enzymes involved in odorant transport and metabolism, respectively. The odorant transporters are mainly represented by odorant binding proteins (OBPs), which belong to the lipocalin protein family [3]. OBPs have been characterized in the nasal mucosa of various species, including rats and humans [4,5]. Additionally, OBPs have been shown to reversibly bind odorants and have been proposed to facilitate odorant access to olfactory receptors. OBPs have also been described as putatively involved in odorant delivery to the cellular cytoplasm for further metabolism [6]. Previous studies have also demonstrated that various enzymes present in the olfactory mucus can metabolize odorants and consequently participate in signal termination and signal modulation if the metabolites are able to activate the receptors. To allow the olfactory receptor to detect iterative signals, odorant molecule elimination appears to be essential [7]. These enzymes that accomplish odorant biotransformation are generally divided into two phases, with the first phase consisting of the functionalization step through oxidation, hydrolysis or reduction of the molecule and the second phase consisting of the conjugation of the phase I metabolite with hydrophilic compounds. Already functionalized molecules can be directly conjugated in phase II, bypassing phase I. Concerning the enzymes belonging to the first phase, electrophysiology recordings of olfactory neurons showed that the inhibition of rat cytochrome P450 monooxygenases increased the electro-olfactogram response amplitude, suggesting a role for these enzymes in signal termination [8]. In mice, it was recently shown that an odorant metabolite resulting from the cytochrome-dependent metabolism of an odorant was able to activate an olfactory receptor [9]. In addition, *ex vivo* real-time recordings of nasal odorant metabolism in rats demonstrated a fast release of volatile metabolites in the tissue headspace [10]. In a heterologous system, a mouse carboxyl esterase was shown to increase or decrease the olfactory receptor response in a specific manner for both the olfactory receptors and the odorants [11]. Concerning phase II enzymes, a pioneer study showed that odorant glucuronoconjugation catalyzed by UDP-glucuronosyl transferases abolished their stimulus properties [12]. Accordingly, electrophysiology experiments showed that the most efficiently glucuronoconjugated odorants triggered the lowest olfactory response [8,13].

Regarding odorant metabolism, another class of phase II enzymes is notable. In pioneering studies, glutathione transferase (GST) activities were measured in the rat OE toward a classical GST substrate: 1-chloro-2,4-dinitrobenzene (CDNB) [14,15]. CDNB was shown to be glutathione conjugated by all classes of GST and absorb the light at 340 nm when conjugated with glutathione. Nineteen GSTs were identified in the rat genome [16], showing either a function of xenobiotic binding [17] or glutathione conjugation activity [18]. Indeed, GSTs promote the conjugation of glutathione (GSH) to a variety of hydrophobic compounds with electrophilic centers, but they are also involved in isomerization reactions [19], glutathione peroxidase activity [20] and simple binding capacity without catalyzing any enzymatic activity. This last function is also called ligandin (with non-substrate ligands) [17,21]. Consequently, GSTs are involved in various biological functions, including detoxification, amino acid catabolism and steroid hormone production. Moreover, extensive evidence supports an additional role in chemo-perception in various species [22]. In mammals, glutathione transferase (GST) was pinpointed as an essential player of the young rabbit responsiveness to the mammary rabbit pheromone [23]. In neonate rabbits, GST was proposed to keep the olfactory receptors sensitive to the mammary pheromone by catabolizing this molecule. Despite this important role in olfaction, only one study has attempted to purify GSTs using nasal epithelium and test their activity toward odorants [15]. Additionally, different GST classes (GSTA, GSTM and GSTP) were detected in the rat OE using immunohistochemistry and antibodies raised against human liver extract. GSTs of the mu class were shown to be the most abundant in the support cells of the OE [24,25]. mRNA expression of this particular class was also confirmed by *in situ* hybridization in the supporting cells of the rat OE [26]. In the present study, we used a combination of experimental approaches to identify and localize GST isoforms in olfactory tissues, including the nasal mucus. Moreover, the interaction of odorants with GSTs was studied *in vitro* using a recombinant GST identified in both tissue and mucus.

## Materials and methods

### Animals

Male weanling specific-pathogen-free Wistar rats (80–100 g) were housed in polypropylene cages and maintained under controlled conditions of a 12-h light-dark cycle, a temperature of 22°C, and 55% humidity for 7 weeks. The rats had free access to water and were fed *ad libitum* with standard food. For measurements, we used a total of 14 rats that were sacrificed by decapitation. The local, institutional and national guidelines and regulations regarding the applied methods, the care and experimental uses of the animals were followed. Thus, all experimental protocols were conducted in accordance with ethical rules enforced by French law and were approved by the local Ethical Committee of the University of Burgundy (Comité d’Ethique de l′Expérimentation Animale Grand Campus Dijon; C2EA grand campus Dijon N°105) and by the French Ministère de l′Education Nationale, de l′Enseignement Supérieur et de la Recherche under the no. 3504. A guidelines checklist is included (S1 Checklist).

### Nasal mucus sampling for mass spectrometry analysis

Wistar male rats were sacrificed by decapitation at 7 weeks. After animal decapitation, 20 μl of sterile filtered PBS at pH 7.0 was introduced into each nostril to increase the mucus volume and facilitate aspiration during the next step. The nasal mucus was then carefully drawn through a sterile tip from each of three different animals.

### Mass spectroscopy analysis

Proteins were separated by sodium dodecyl sulfate polyacrylamide gel electrophoresis (SDS-PAGE) and digested with trypsin. An UltiMate 3000 rapid separation liquid chromatography (RSLC) Nano System (Thermo Fisher Scientific, Waltham, USA) was used for separation of the protein digests. Peptides were automatically fractionated onto a commercial C18 reversed-phase column (75 μm × 250 mm, 2-μm particle, PepMap100 RSLC column, Thermo Fisher Scientific (Waltham, USA), temperature 35°C). Trapping was performed for 4 min at 5 μL/min with solvent A (98% H_2_O, 2% acetonitrile and 0.1% formic acid). Elution was performed using two solvents, A (0,1% formic acid in water) and B (0.1% formic acid in acetonitrile), at a flow rate of 300 nL/min. Gradient separation was 3 min at 3% B, 110 min from 3% to 20% B, 10 min from 20% to 80% B, and maintained for 15 min. The column was equilibrated for 6 min with 3% buffer B prior to the next sample analysis. The eluted peptides from the C18 column were analyzed by Q-Exactive instruments (Thermo Fisher Scientific, Waltham, USA). The electrospray voltage was 1.9 kV, and the capillary temperature was 275°C. Full mass spectrometry (MS) scans were acquired in the Orbitrap mass analyzer over the m/z 300–1200 range with a resolution of 35,000 (m/z 200). The target value was 3.00E+06. The fifteen most intense peaks with charge states between 2 and 5 were fragmented in the higher-energy collisional dissociation cell (HCD) with a normalized collision energy of 27%, and the tandem mass spectrum was acquired in the Orbitrap mass analyzer with a resolution of 17,500 at m/z 200. The target value was 1.00E+05. The ion selection threshold was 5.0E+04 counts, and the maximum allowed ion accumulation times were 250 ms for full MS scans and 100 ms for tandem mass spectra. Dynamic exclusion was set to 30 s.

### Raw data analysis

Raw data collected during nanoLC-MS/MS analyses were processed and converted into *.mgf peak list format with Proteome Discoverer 1.4 (Thermo Fisher Scientific, Waltham, USA). MS/MS data were interpreted using the search engine Mascot (version 2.4.0, Matrix Science, London, UK) installed on a local server. Searches were performed with a tolerance on mass measurement of 0.2 Da for precursor and 0.2 Da for fragment ions against a composite target decoy database (16118 total entries) built with the *Rattus norvegicus* Swissprot database (TaxID 10116—September 25, 2019–7941 entries) fused with the sequences of recombinant trypsin and a list of classical contaminants (118 entries). Cysteine carbamidomethylation, methionine oxidation, protein N-terminal acetylation and cysteine propionamidation were searched as variable modifications. Up to one trypsin and missed cleavage was allowed. For each sample, peptides were filtered out according to the cut-off set for protein hits with one or more peptides taller than nine residues, ion score >20, identity score >8, and 1% false positive rate.

### Preparation of subcellular olfactory epithelium (OE) fractions for Western blot analysis

Animals were sacrificed by decapitation. Careful OE dissection was performed to avoid contamination with respiratory epithelium. The freshly removed OE was immediately stored at -80°C until extraction. Pools of eight OE were homogenized in 3 mL of 10 mM Tris-HCl buffer (pH 7.4) containing 0.15 M sucrose using an Ultra-Turrax homogenizer. The homogenate was centrifuged at 105 000 g for 25 min to separate soluble and insoluble fractions. The supernatant fraction obtained following this step was designated the cytosolic fraction (S1). The resulting pellet was resuspended in 500 μL lysis buffer (NP40 lysis buffer from Life Technologies, Carlsbad, USA) with 0.1% SDS, (0.5%, w/v) sodium deoxycholate, 1 mM phenylmethylsulfonyl (PMSF) and protease inhibitor cocktail to solubilize membrane proteins. The new homogenate was sonicated for 20 s, incubated for 1 h at 4°C under strong agitation using a Vortex Genie II mixer (Scientific Industries, Bohemia, USA) and then centrifuged at 105 000 g for 30 min at 4°C. The resulting supernatant was designated the solubilized membrane protein fraction (S2). The last insoluble pellet was resuspended in lysis buffer (fraction C). All fractions were stored in small aliquots at -80°C until use. The protein levels of these fractions were quantified by the Lowry method using a DC protein assay (Bio-Rad, Hercules, USA) and bovine serum albumin as a standard.

### Western blot analysis

The samples (60 μg/well) were loaded onto 4–15% SDS-PAGE gels. The molecular weight marker (Precision Plus Dual Xtra Standards, Bio-Rad, Hercules, USA) was loaded into the first lane of each gel. SDS-PAGE was performed using a Mini-Protean II system (Bio-Rad, Hercules, USA). Following electrophoresis at 200 V for 30 min, the proteins were transferred to polyvinylidene fluoride membranes (Immun-Blot PVDF, Bio-Rad, Hercules, USA) at 100 V for 10 min with a Trans-Blot Turbo Transfer System (Bio-Rad, Hercules, USA). The membranes were blocked in a solution containing 10 mM Tris-HCl pH 8.0, 150 mM NaCl, 0.05% Tween 20 and 5% nonfat dry milk (TBST) for 1 h at room temperature. The blots were then incubated overnight at 4°C with the primary antibodies rabbit anti-GSTM1 (PAA658Ra01), rabbit anti-GSTM2 (PAA657Hu01) or rabbit anti-GSTM4 (PAA643Ra01) from Cloud-Clone Corp or with rabbit anti-GSTA1 (ab180650) from Abcam diluted at 1/500 in TBST (anti-GSTA1 were purchased from Abcam (Cambridge, UK) ab180650; all GST mu antibodies were purchased from Cloud-Clone Corp. (Katy, USA). After washing (five 5-min washes with TBST), the membranes were incubated with goat anti-rabbit horseradish peroxidase-conjugated secondary antibody (diluted 1:25000) for 1 h at room temperature and then rinsed five times with TBST. The protein-antibody complexes were detected using an ECL chemiluminescence kit (Clarity Western ECL Substrate, Bio-Rad, Hercules, USA) and a ChemiDoc XRS Imaging System (Bio-Rad, Hercules, USA).

### Immunohistochemistry experiments

Rat heads (three) were fixed with 4% formaldehyde in phosphate-buffered saline (PBS, 0.1 M pH 7.4, Sigma-Aldrich) for 48 h at room temperature. After decalcification with 10% Titriplex III (ethylenediaminetetraacetic acid disodium salt, Sigma-Aldrich, Saint-Louis, USA) in PBS pH 7.4 for two weeks with daily changes of this solution, the specimens were dehydrated through a series of alcohol and toluene baths and then embedded in paraffin. Five-micrometer-thick sections were deparaffinized, rehydrated and stained immunohistochemically. An antigen pretreatment step was carried out using high-temperature antigen unmasking techniques with target retrieval in citrate buffer pH 6.0 for 40 min. Endogenous peroxidases were treated with blocking reagent (Dako, Santa Clara, USA) for 10 min at room temperature prior to equilibration in 0.05 M Tris-HCl, 0.15 M NaCl, and 0.05% Tween 20 at pH 7.6. Tissue sections were saturated for 45 min with 10% goat serum (Sigma-Aldrich, Saint-Louis, USA) in antibody diluent (Dako, Santa Clara, USA) to reduce nonspecific binding. Incubation in the primary antibody was performed for 90 min at room temperature (1:50 for anti-GSTA1, 1:400 for anti-GSTM1, 1:150 for anti-GSTM2 and 1:400 for anti-GSTM4). Negative controls were prepared by replacing the primary antibody with antibody diluent alone. Tissue sections were subsequently incubated for 45 min at room temperature in a 1:200 dilution of the secondary antibody in antibody diluent (goat anti-rabbit coupled with horseradish peroxidase (HRP) purchased from Dako, Santa Clara, USA, P0448). Immunohistochemical staining was performed using a liquid 3,3′-diaminobenzidine (DAB) + substrate chromogen system (Dako, Santa Clara, USA). Sections were counterstained with Mayer’s hemalum solution (Merck, Kenilworth, USA). The slides were examined with an Eclipse E600 microscope equipped with Plan Fluor objectives. Images were acquired with a DS-Ri2 digital camera using the software NIS-Elements Basic Research (all from Nikon, Tokyo, Japan).

### GSTM2 production and purification

The DNA sequence encoding the *Rattus norvegicus* GSTM2 (UniProt code P08010) was optimized for expression in *E*. *coli*, synthesized by Genewiz (Leipzig, Germany), and subcloned into the pET22b vector (Novagen, Darmstadt, Germany) between the *NdeI* and *SacI* restriction sites. The optimized sequence with the additional insertion of the two restriction sites is provided (S1 Appendix). During DNA optimization, the sequence GC content decreased from 51.5% to 50.5%, and all of the codons were adapted to change it by the most occurring codon following the *E*. *coli* codon usage table. The resulting plasmid was subsequently transformed into the *E*. *coli* BL21 (DE3) strain (Novagen, Darmstadt, Germany). The expression of recombinant GSTM2 was performed at 37°C in Luria Bertani (LB) medium supplemented with 100 μg.mL^-1^ ampicillin. When the cell culture reached an OD_600 nm_ of 0.6, recombinant protein expression was induced by the addition of 1 mM isopropyl β-D-1-thiogalactopyranoside (IPTG), and the cells were grown for an additional 24 h at 37°C. The cells were then harvested by centrifugation, suspended in binding buffer (PBS buffer, pH 7.0), disrupted at 4°C through two 3 min sonication cycles and finally centrifuged at 20 000 g for 45 min at 4°C. The supernatant of the cell lysate was loaded onto a GST Trap Fast Flow 5 mL column (GE Healthcare, Chicago, US). The column was washed with five column volumes of binding buffer before injecting the protein sample, and the recombinant GSTM2 protein was eluted using a 50 mM Tris-HCl pH 7.4 buffer supplemented with 10 mM glutathione. The fractions containing the recombinant GSTM2 were pooled, incubated with 100 mM dithiothreitol (to reduce the free Cys residue present in the protein) for 15 min and further dialyzed against a 100 mM potassium phosphate pH 6.5 buffer. GSTM2 was finally concentrated to 10 mg.mL^-1^ using a Vivaspin concentrator with 10 kDa of membrane cut-off (Startorius, Göttingen, Germany) and stored at -20°C. The purity of GSTM2 was evaluated using SDS-PAGE [27]. The molecular concentration was determined spectrophotometrically using an extinction coefficient of 20 400 M^-1^·cm^-1^ at 280 nm.

### Inhibition GST assay

Stock solutions of odorants were prepared in methanol at a concentration of 10 or 100 mM. All solutions were stored at -20°C. The tested odorants include limonene oxide, (+) limonene oxide, (-) limonene oxide, 2,4,6-trichloroanisole, 2-butanethiol, 6-methyl-5-hepten-2-one, beta-ionone, cinnamaldehyde, citral, cyclohexanone, dimethyl disulfide, geraniol, geranyl acetate, hexanoic acid, mesityl oxide, R-carvone, S-carvone, trans-2-nonenal, trans-2-hexen-1-al and vanillin. The slope of the initial rate of the reaction was measured in the absence or presence of odorants (10 μM or 100 μM). For each measurement, the same mixture containing buffer, glutathione and 1-chloro-2,4-dinitrobenzene (CDNB) was used to fill six cuvettes and supplemented independently with an odorant in three cuvettes or an equivalent volume of methanol in the three others. Then, the reaction is started and immediately measured by adding the same amount of enzyme in each cuvette sequentially. The reactions were performed at 20°C in 1 mL of 100 mM potassium phosphate buffer at pH 6.5 in the presence of 1 mM GSH and 1 mM CDNB. Recombinant GSTM2 (5 nM) was added, and the absorbance increase was measured using a V-730 spectrophotometer (Jasco, Tokyo, Japan). The inhibition percentage was calculated using the following equation: Inhibition percentage = (slope of the initial rate (with odorant)/slope of the initial rate (without odorant) * 100, where the slope of the initial rate was obtained from the average of three measurements that were obtained as described for the activity assay. Statistical analysis was performed using a bilateral Student’s t-test. Asterisks indicate statistical significance (*P < 0.05 and **P < 0.01).

### Assessment of enzymatic glutathione conjugation

Enzymatic incubations were carried out in a 50 μl reaction mix containing GSTM2 (100 μM), GSH (15 mM) and odorant (300 mM) in PBS. After 80 min incubation at 37°C, the reaction was stopped by adding 50 μl of a 25% CuSO_4_ solution and centrifuged for 10 min at 14 000 g. The supernatants containing glutathione-odorant conjugates were diluted in two volumes of ultrapure water. Then, the dilution was analyzed by high-performance liquid chromatography (HPLC) (Ultimate 3000) equipped with a dual low-pressure gradient pump with vacuum degasser, a thermostated autosampler (14°C), a thermostated (set to 30°C) Hypersil GOLD C18 analytical column (150 mm, 2.1 mm; 3-μm particle size; Thermo Scientific, Waltham, USA) and a Corona Ultra RS Charged Aerosol Detector (CAD; Thermo Scientific Dionex, Sunnyvale, USA). The nebulizer temperature was set to 14°C. The response range was set to 100 pA full scale.

The analysis was performed using a multistep gradient with (A) 0.1% trifluoroacetic acid in ultrapure water and (B) 0.1% trifluoroacetic acid in methanol as the mobile phase. Gradient elution started at 99.5% (A) and 0.5% (B) for 6 min followed by an increase to 100% (B**)** at 21 min and then a reduction to 99.5% (A) and 0.5% (B) at 25 min for 3 min. The flow rate of the mobile phase was set at 0.6 mL.min^-1^ during the 28 min analysis time, and the injection volume was 5 μl of sample. Data processing was carried out with Chromeleon 7.2 software (Dionex, Sunnyvale, USA), and the peak area (pA.min^-1^) corresponding to glutathione-odorant conjugate was integrated. To determine the enzymatic glutathione conjugation of odorants, HPLC quantifications of the odorant conjugates were systematically performed in the presence and absence of recombinant GSTM2, two conditions representing the total conjugation (enzymatic and non-enzymatic) and the non-enzymatic conjugation, respectively. Enzymatic glutathione conjugation was defined by subtracting the non-enzymatic conjugation part from the total conjugation resulting from three independent measures. Statistical analysis was performed using a bilateral Student’s t-test. Asterisks indicate statistical significance (*P < 0.05 and **P < 0.01).

## Results

### Identification of the rat proteins in the nasal mucus

**Fig 1:**
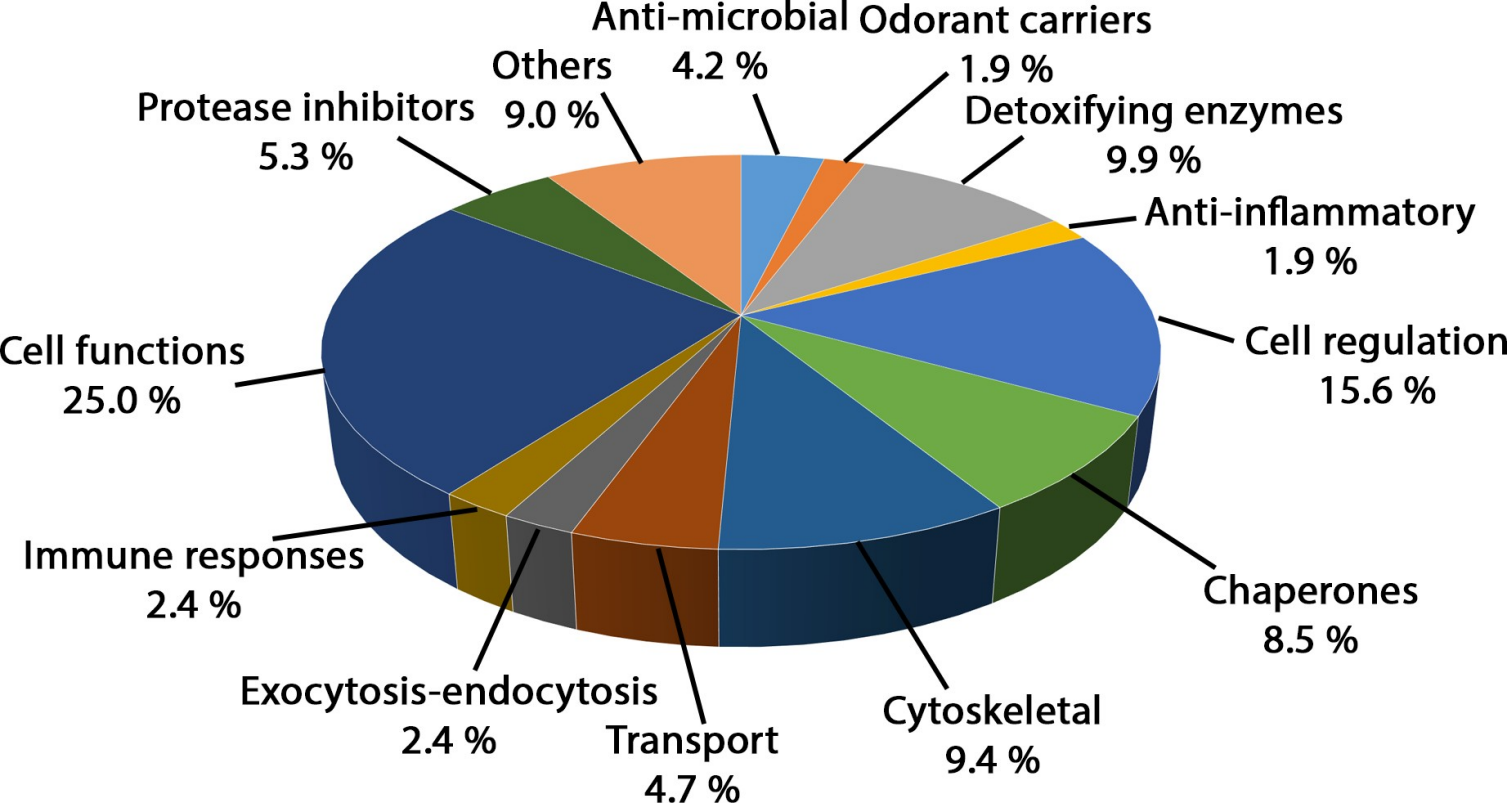
Functional classification of the total rat nasal mucus. The 211 proteins identified with more than one peptide were classified into 13 functional categories. For each category, a percentage is indicated. Each protein is counted in only one category.

Three different samples from three adult male rats were used to identify 364 proteins in the nasal mucus, which are presented in S1 Table. A total of 211 proteins were identified with more than one peptide in at least one of the three rat nasal mucus proteomes and were classified into 13 different functional categories (Fig 1). Although some proteins could have been included in different categories, they were counted in only one category. A total of 25.0% of the proteins were involved in cell function, such as ribosomal constituents, or enzymes that play a role in the citric acid cycle, such as isocitrate dehydrogenase. Closely related to this group, another group consisting of 15.6% of the identified proteins were involved in the regulation of cell functions (e.g., ADP-ribosylation factors). The proteins involved in the cytoskeleton itself or the machinery linked to the cytoskeleton formed another well-represented group with 9.4% of the total proteins. For example, tubulin, keratin and clathrin, which shape rounded vesicles in the cytoplasm, were in this group. As previously observed in mice and human mucus proteomes, many proteins involved in bacterial resistance were identified [1,2]. These proteins can be involved in this resistance directly, such as proteases or ribonucleases (4.2%), or indirectly, such as the group of proteins consisting of protease inhibitors (5.2%). Another group (8.5%) comprised chaperone proteins, such as heat shock proteins 70 and 90. These proteins present different functions, including refolding and anti-aggregation. Two groups of proteins were particularly interesting in the context of olfaction, the proteins involved in the transport of odorants and the enzymes involved in detoxification, and these two important groups represented 4.7 and 9.9% of the total protein identified, respectively.

### Identification of the rat proteins potentially involved in odorant transport and metabolism

Concerning the proteins that are able to interact with odorants, one OBP, OBP1F, was identified in the three tested rats with 5 different peptides and with a large number of spectra (S1 Table). Interestingly, two other proteins of the same lipocalin family that potentially act as odorant carriers were identified: neutrophil gelatinase-associated lipocalin (P30152) and the major urinary protein (P02761). Vomeromodulin (Q63751), a putative pheromone carrier protein, was identified, as previously reported [28].

**Fig 2:**
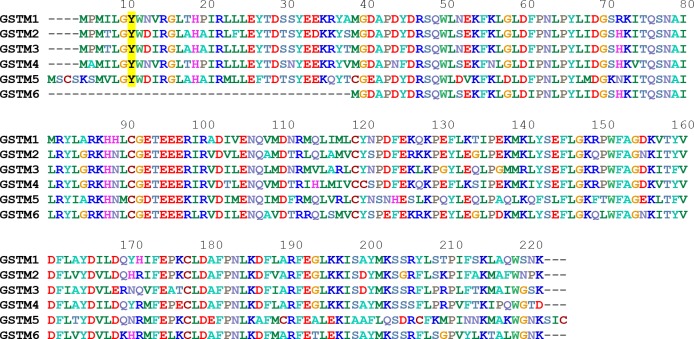
Sequence alignment of the mu class of rat GSTs. The numbering of the amino acid residues is based on the GSTM5 sequence. The sequences were aligned with Bioedit software. The amino acids are colored based on their physicochemical properties. The catalytic Tyr residue is indicated in black and highlighted in yellow. The accession numbers for GSTM1, 2, 3, 4, 5 and 6 are P04905, P08010, P08009, B29231, Q9Z1B2 and A0A0G2K6L4, respectively.

Concerning the 21 identified enzymes that are linked to detoxification, 6 are directly involved in oxidative stress resistance, such as catalase or peroxiredoxin (Table 1). The other 15 may be involved in odorant metabolism. Odorants present a large chemical diversity with different chemical functions, such as alcohols, aldehydes and ketones, without being exhaustive. In this context, enzymes targeting these chemical functions and located in the nasal mucus can potentially catalyze the biotransformation of these odorants. Interestingly, 3 aldehyde dehydrogenases (P11883, P13601 and P11884) and 2 alcohol dehydrogenases (P41682 and P51635) were identified. Aldehyde dehydrogenases were previously shown to be involved in the insect odorant metabolism [29]. Another enzyme, sulfotransferase (P50237), which was previously shown to be putatively involved in mouse odorant metabolism, was also identified [30]. Additionally, three GSTs were identified: GSTM2 (P08010), GSTM1 (P04905) and GSTP1 (P04906). Among these GSTMs, GSTM2 presence was confirmed in the three proteomes from the three studied rats and appears with the most identified spectrum. GSTM1 was identified in two of the three proteomes, and GSTP1 was identified in only one of the proteomes. Analysis of proteins identified with only one identified peptide (excluded from Fig 1) also revealed the presence of additional GSTs: GSTA2 (P04903), GSTA4 (P14942) and GSTA1 (P00502). GSTA1 and GSTA2 were identified with a unique and common peptide and do not allow us to conclude whether both enzymes or only one are expressed. Three of the 6 predicted GSTMs based on genome analysis were identified in the rat nasal mucus (Fig 2). The different GSTMs have a high percent amino acid identity, showing the close relationship of these proteins belonging to the mu family (Table 2).

**Table 1:** Identification of rat nasal mucus enzymes involved in detoxification.

Enzyme name	Accession number	Number of identified peptides (number of spectra) in rat 1, 2 and 3
Aldehyde oxidase 4	Q5QE79	25 (38), 3 (3), 2 (2)
Retinal dehydrogenase 1	P51647	8 (11), 6 (9), 1 (1)
Glutathione peroxidase 6	Q64625	5 (16), 2 (4), 0 (0)
Glutathione S-transferase M2	P08010	12 (15), 3 (3), 1 (1)
Aldehyde dehydrogenase, dimeric NADP-preferring	P11883	5 (9), 2 (3), 5 (7)
Carbonic anhydrase 1	B0BNN3	3 (7), 6 (11), 0 (0)
Sulfotransferase 1C1	P50237	8 (10), 2 (3), 0 (0)
Peroxiredoxin-5	Q9R063	5 (5), 3 (4), 0 (0)
Peroxiredoxin-6	O35244	6 (7), 2 (2), 0 (0)
Glutathione S-transferase M1	P04905	7 (7), 1 (1), 0 (0)
Carbonic anhydrase 2	P27139	3 (3), 4 (5), 0 (0)
Peroxiredoxin-2	P35704	4 (6), 2 (2), 1 (1)
Glutathione S-transferase P1	P04906	2 (6), 0 (0), 0 (0)
Peroxiredoxin-1	Q63716	3 (3), 2 (2), 0 (0)
Aldehyde dehydrogenase, cytosolic 1	P13601	3 (3), 0 (0), 0 (0)
Alcohol dehydrogenase class 4 mu/sigma	P41682	3 (3), 0 (0), 0 (0)
Aldehyde dehydrogenase, mitochondrial	P11884	3 (3), 0 (0), 0 (0)
Superoxide dismutase [Cu-Zn]	P07632	0 (0), 3 (3), 0 (0)
Catalase	P04762	0 (0), 2 (2), 0 (0)
3-alpha-hydroxysteroid dehydrogenase	P23457	2 (2), 0 (0), 0 (0)
Alcohol dehydrogenase [NADP(+)]	P51635	2 (2), 0 (0), 0 (0)

For each protein, the accession number is indicated. The number of identified peptides and spectra recorded from each of the three analyzed rats is shown.

**Table 2:** Amino acid sequence identity within the GSTs of the mu class.

	GSTM1	GSTM2	GSTM3	GSTM4	GSTM5	GSTM6
GSTM1	100	78	78	82	62	64
GSTM2		100	80	76	64	72
GSTM3			100	75	65	64
GSTM4				100	56	64
GSTM5					100	53
GSTM6						100

The percent identity value was calculated for each pair of sequences after alignment of the two sequences.

### Western blot analysis revealed the presence of a GST belonging to the mu class in the rat OE

**Fig 3:**
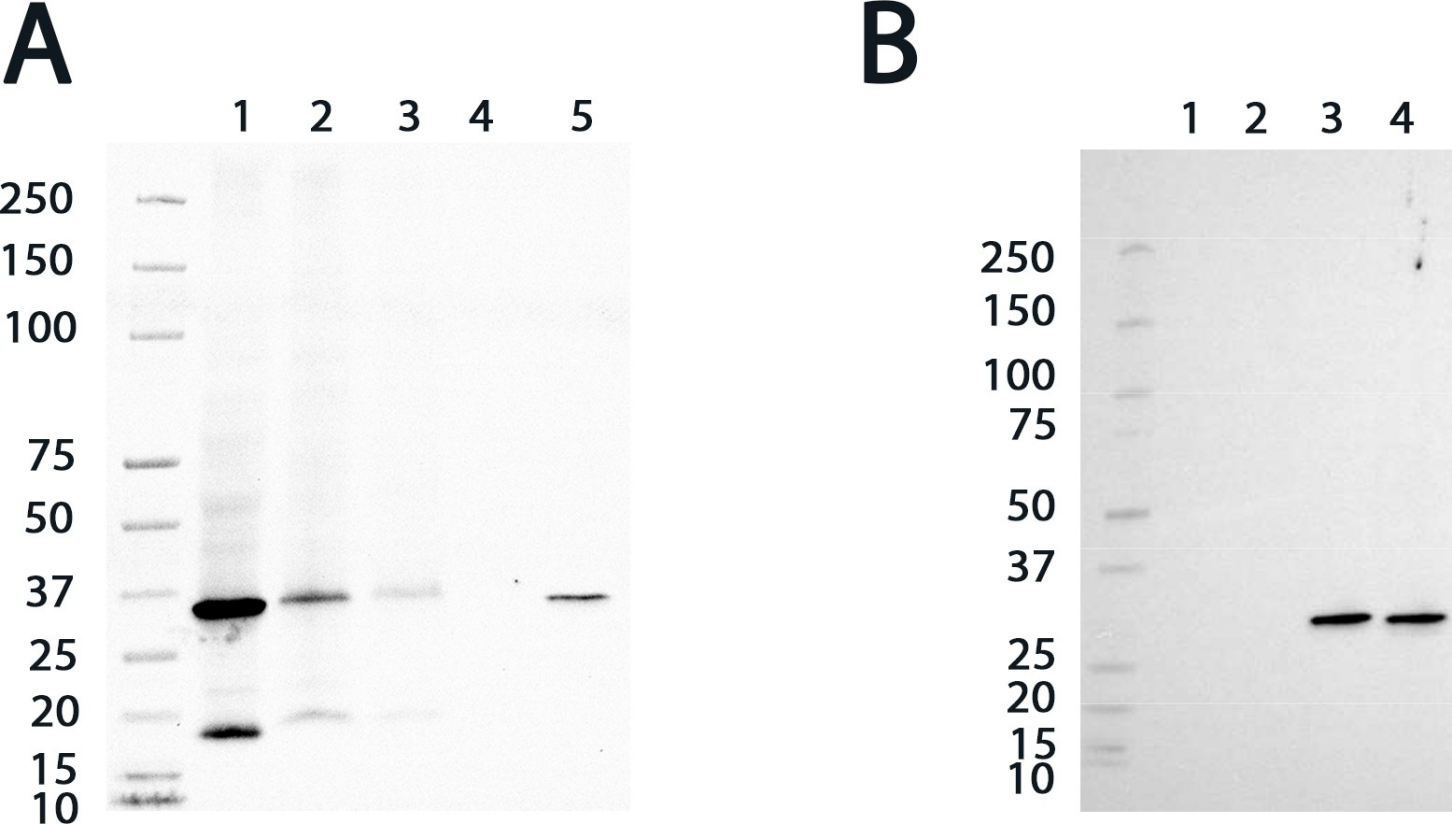
Western blot analysis of GSTM2 in rat OE. (A) GSTs of the mu class were detected in two fractions of the supernatant of the OE lysate (extract S1 for lane 1 and extract S2 for lane 2) and in the pellet of the OE lysate (extract C for lane 3). Lanes 4 and 5 correspond to the nasal mucus and the purified recombinant GSTM2 (100 ng) from the rat, respectively. The molecular weight markers are indicated on the left of the gels in kDa. (B) Western blot analysis using the GSTM2 antibody shows the absence of the detection of recombinant GSTA1 (100 ng) and GSTP1 (100 ng) in lanes 1 and 2 but the detection of human recombinant GSTM1 (100 ng) and rat recombinant GSTM2 (100 ng) in lanes 3 and 4, respectively.

**Fig 4:**
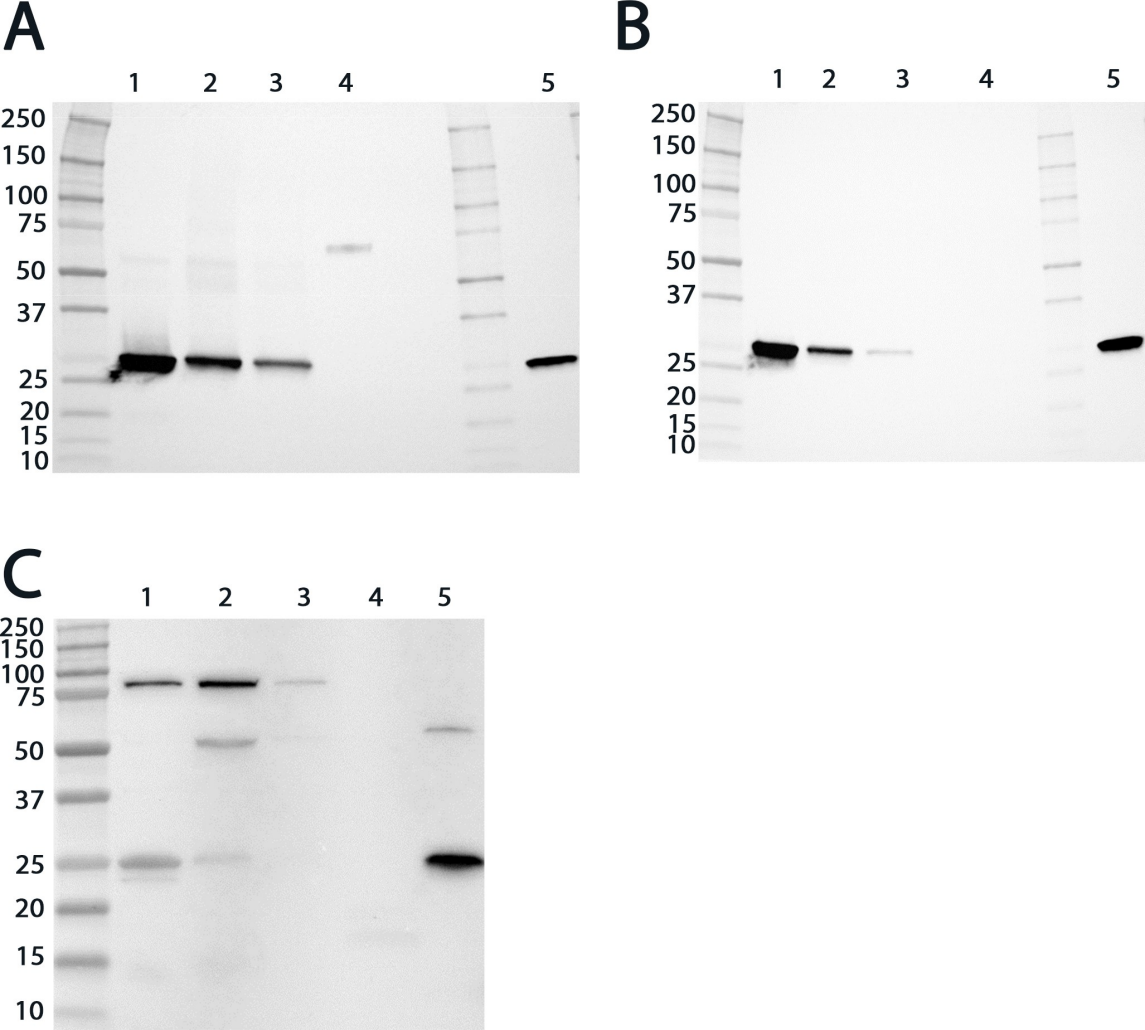
Expression analysis of rat GSTs of the mu and alpha classes. (A) Western blot analysis of rat GST expression using antibodies against GSTM1, (B) GSTM4 and (C) GSTA1. The different blots include two fractions of the supernatant of the OE lysate (extract S1 for lane 1 and extract S2 for lane 2, all panels), the pellet of the OE lysate (extract C for lane 3, all panels), the nasal mucus (lane 4) and the purified recombinant rat GSTM2 (100 ng) (lane 5, panel A and B) or the purified recombinant human GSTA1 (100 ng) (lane 5, panel A). The molecular weight markers are indicated on the left of the gels in kDa.

Based on the proteomic analysis showing many spectra identified belonging to the mu class, namely, GSTM2, a Western blot analysis of the OE and mucus was conducted. As a positive control, we confirmed that the GSTM2 antibody bound to the recombinant rat GSTM2 (Fig 3A, line 5 and Fig 3B, line 3). However, this antibody bound to recombinant human GSTM1 (Fig 3B, line 4) but not recombinant human GSTA1 and GSTP1 (Fig 3B, lines 1 and 2). These results suggest a specific binding toward the mu class GST (GSTMs) but do not enable the members of the mu class to be distinguished. Two other additional antibodies were tested: one directed toward GSTM1 and another directed toward GSTM4 (Fig 4A and 4B). Both bound to the recombinant rat GSTM2, suggesting again, in keeping with the strong sequence identity of the mu class GSTs, the binding toward all GSTs of the mu class (Fig 2 and Table 2). Therefore, they do not allow specific discrimination of the different GSTMs. Western blot analyses using these three different antibodies revealed the presence of GSTs of the mu class in the supernatant and in the pellet of the OE (Figs 3A, 4A and 4B). The band analysis corresponds to the expected molecular mass of the mu class and was confirmed by migration of the recombinant rat GSTM2 protein to the same level in the gel. The presence of a unique band also supports that the antibody specifically binds the GSTs of the mu class. It also excludes the detection of GSTM6, which has a lower molecular weight and was not detected in the proteomic analysis. The highest intensity was in the supernatant in comparison with the pellet, which supports the presence of GSTMs in the soluble cell fraction. The band observed in the pellet may represent a misfolded part of the GSTMs. The absence of a band in the nasal mucus indicates an absence of either the GSTMs or a low concentration. The low amount of nasal mucus harvested during the sampling does not allow a more concentrated sample to be tested. As a control, an antibody against alpha class GSTs was tested. GSTs of the alpha class were identified in the mucus of only one of the three rats at a low level (one and two spectra, respectively). As expected, the alpha class was poorly detected in the OE and not detected in the olfactory mucus (Fig 4A). This observation supports the potential major role of the GSTs of the mu class among the different GST classes in the OE.

### Rat GSTs of the mu class are expressed in the OE

**Fig 5:**
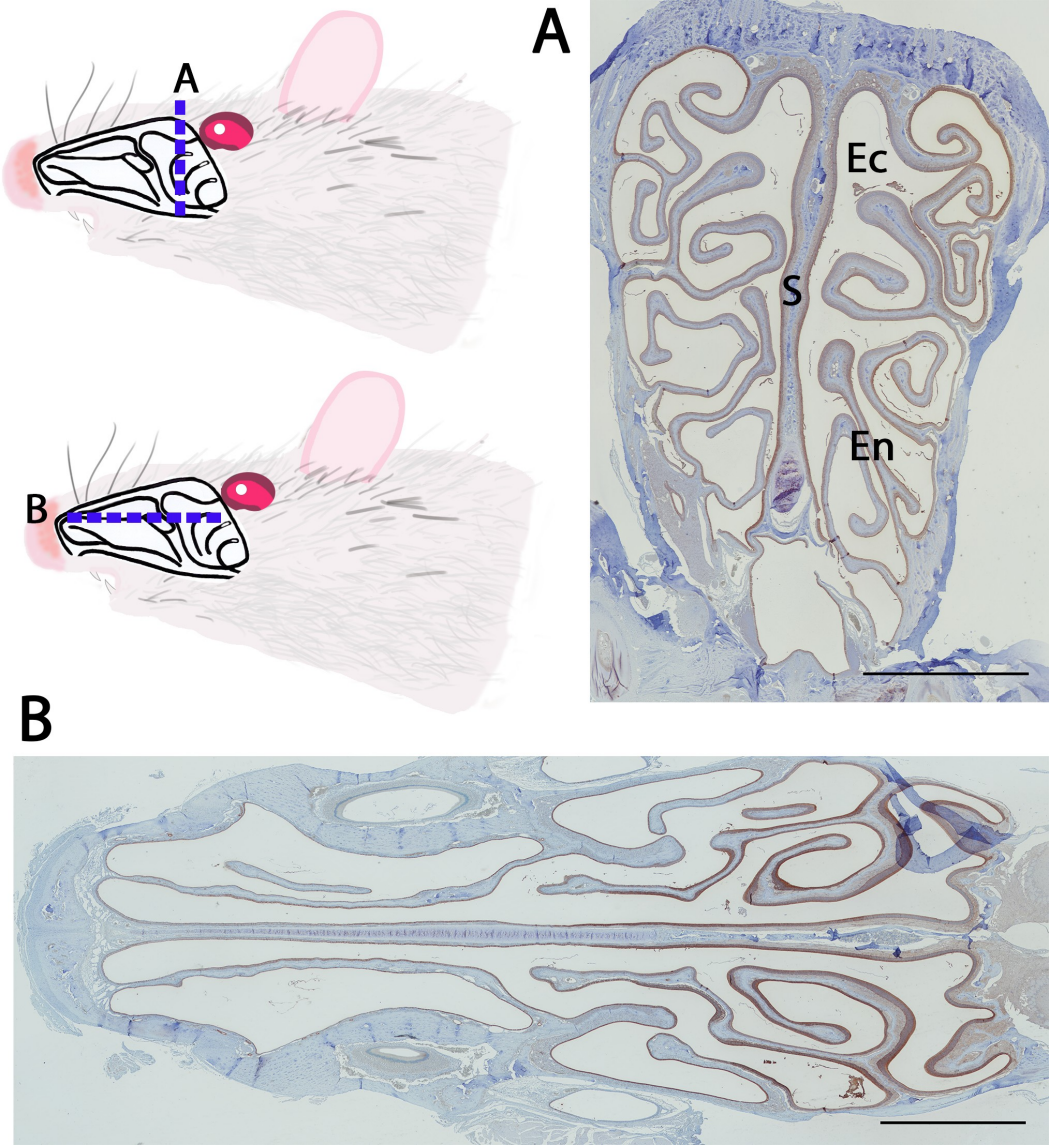
Expression of GSTs of the mu class in the rat nasal cavity. Distribution of the mu class GST immunoreactivity using an anti-GSTM2 antibody. The two sections of the (A) frontal and (B) transverse views of the rat nasal cavity are represented on the rat schema. Ectoturbinates (Ec), septum (S) and endoturbinates (En) are indicated. The scale bar is 2300 μm.

**Fig 6:**
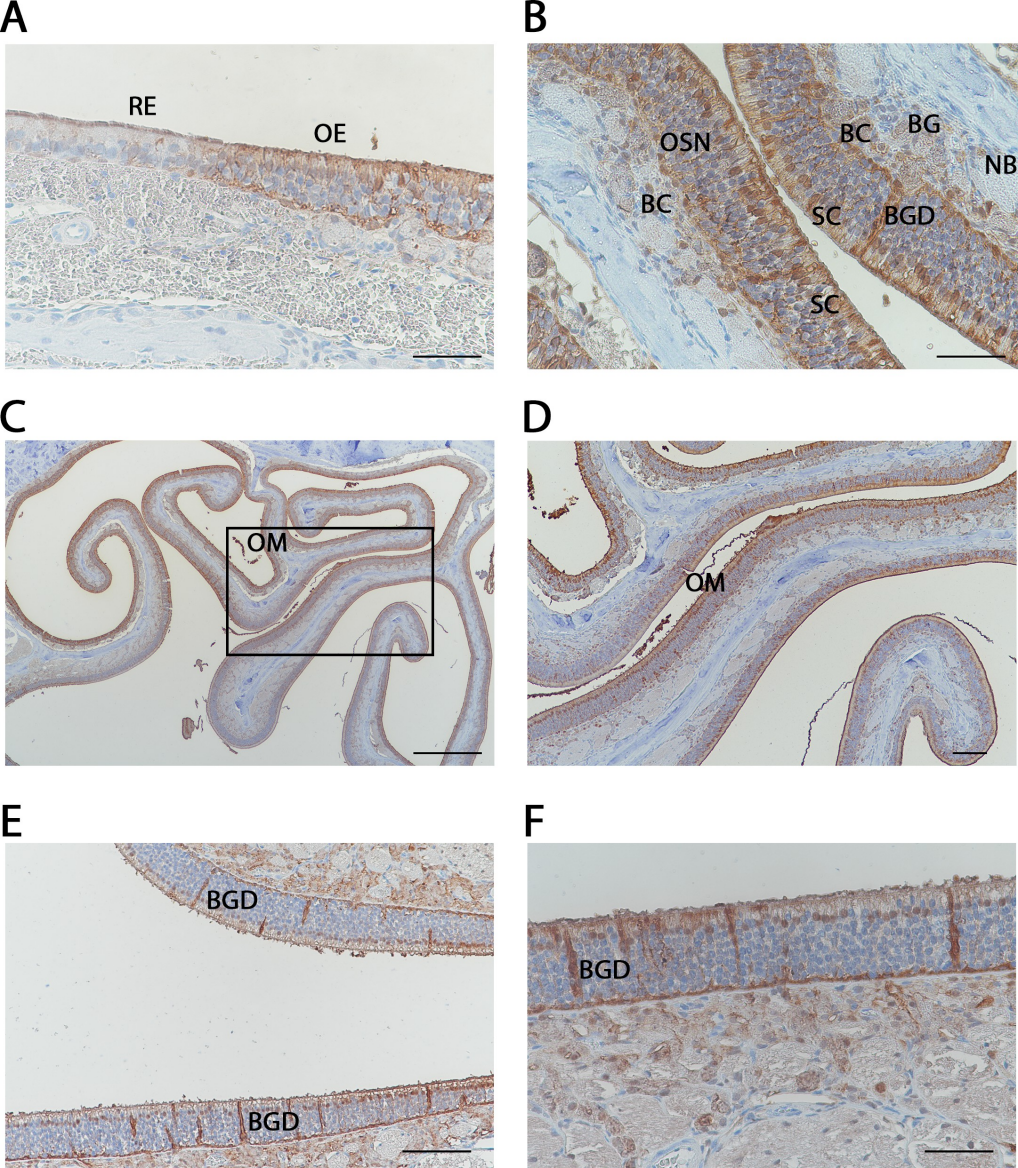
Immunohistochemistry analysis of mu class GST expression. Distribution of the mu class GST immunoreactivity using an anti-GSTM2 antibody. (A, B, C and D) Different details showing the olfactory (OR) and respiratory epithelium (RE) and different cell types and structures, including the olfactory sensory neurons (OSN), sustentacular cells (SC), basal cells (BC), nerve bundle (NB), Bowman’s glands (BG), and Bowman’s gland duct (BGD). Panel D is a higher magnification of panel C, showing the nasal olfactory mucus (OM). The scale bar is 50 μm for panels A, B and F, 100 μm for panel E, 200 μm for panel D and 500 μm for panel C.

We investigated the localization of GSTMs in the nasal cavity of rats. The two views presented in Fig 5A and 5B show a frontal and transverse section of the rat nasal turbinates, respectively. Extensive staining with the antibody against rat GSTMs is observed in the two views. The staining is more intense in the OE compared to the respiratory epithelium (Fig 5B), as previously reported for GSTM1 detected with antibodies raised against liver GSTs [24]. A clear demarcation can be observed between the respiratory and OE, which present a different cellular organization (Fig 6, panel A). A stronger binding toward the mu class GSTs was observed in the OE. Staining was observed in different types of cells, including the olfactory sensory neurons, the sustentacular cells, the basal cells and the nerve bundle (Fig 6, panel B). Characteristic structures of the OE, such as Bowman’s glands (BG) and Bowman’s glands duct (BGD), were also identified (Fig 6, panel B). The tissue preparation allowed us to trap some olfactory mucus inside the nasal turbinates (Fig 6, panel C), which was also presented in an enlarged view (Fig 6, panel D). An intense staining in the olfactory mucus indicates the presence of mu class GSTs. The strong staining of the Bowman’s glands and the associated duct (Fig 6, panels E and F) also supports this observation. Indeed, the Bowman’s gland might secrete proteins in the Bowman’s gland duct.

**Fig 7:**
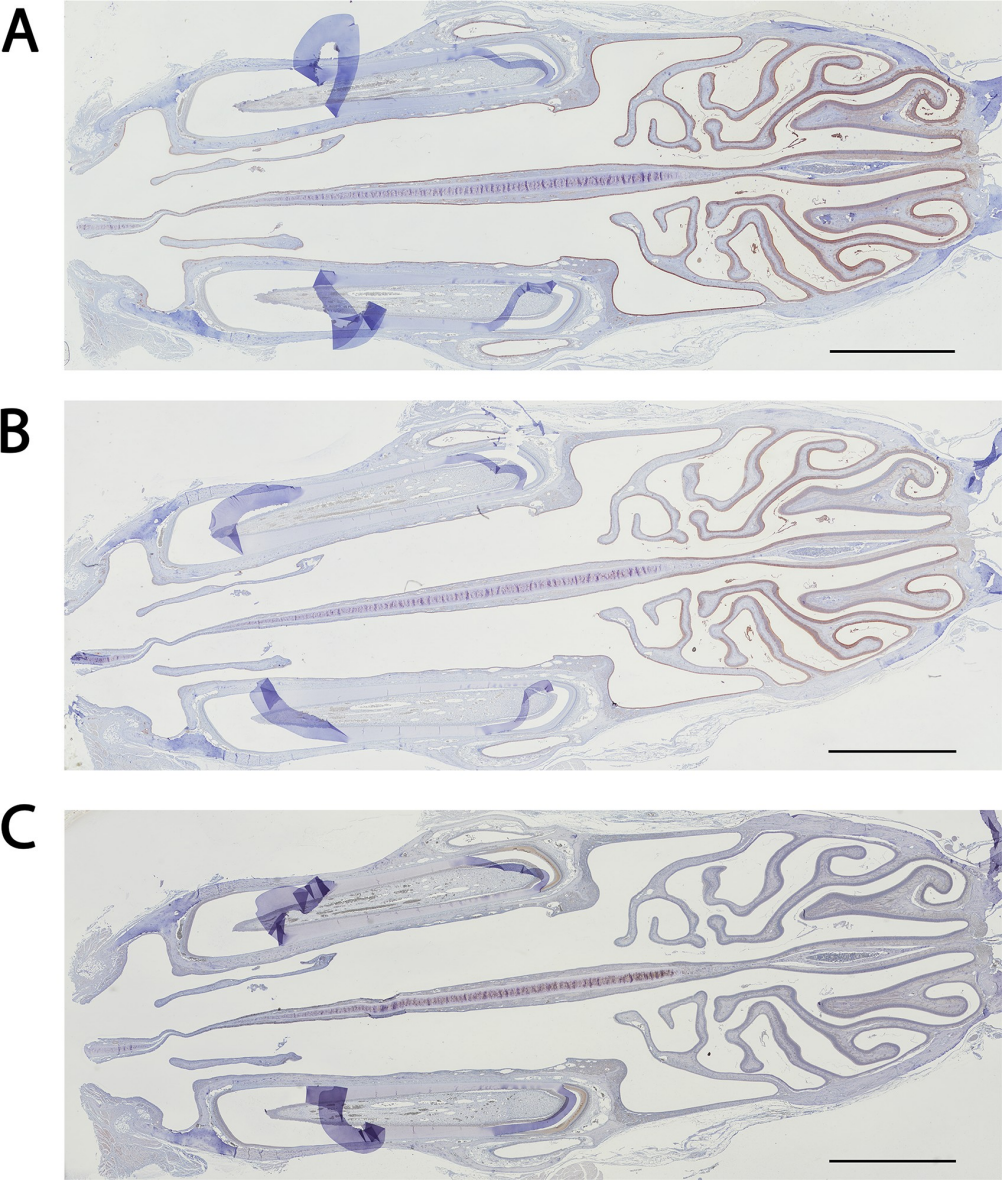
Immunohistochemistry analysis of the expression of the mu and alpha class GSTs. Histological expression pattern of mu class GSTs using anti-GSTM1 (A), GSTM4 (B) and GSTA1 (C) antibodies. The scale bar is 2300 μm for the three panels.

The three antibodies directed against GSTM1, GSTM2 and GSTM4 were used in immunohistochemistry. As expected, considering the Western blot analysis, the same staining was obtained for the three antibodies (Fig 7A and 7B). In addition, poor staining was observed for GSTs of the alpha class, suggesting a lower expression of GSTAs in the OE (Fig 7C).

### Determination of the capability of odorants to inhibit CDNB glutathione conjugation activity by GSTM2

Based on the immunohistochemistry analysis that suggested the presence of GSTs of the mu class in the olfactory mucus, which was reinforced by the presence of these enzymes in the Bowman canal duct, we proposed to biochemically characterize the possible interaction of these enzymes with odorants. We focused our analysis on rat GSTM2, since the mass spectrometry analysis allowed us to identify this particular member of the mu class in the olfactory mucus of three different rats. The rat GSTM2 was produced in *E*. *coli*, and GSTM2 was purified in a single step using a GSTtrap affinity column at more than 99% purity.

**Fig 8:**
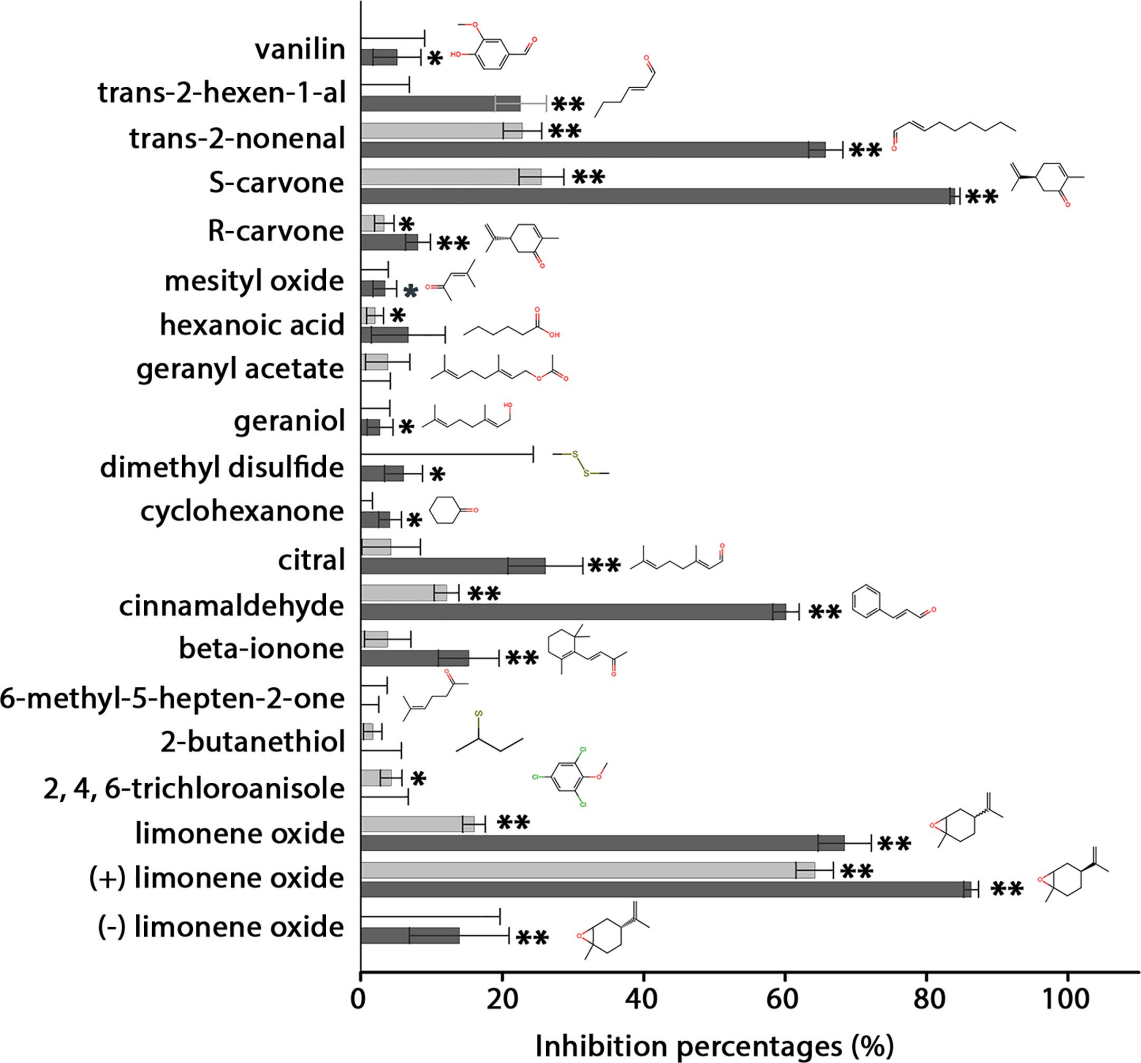
Measure of the inhibition rates of odorant molecules toward GSTM2 activity. Inhibition of recombinant rat GSTM2 by 20 odorant molecules. The inhibition rates for rat GSTM2 acting on CDNB were calculated at a concentration of 1 mM CDNB and glutathione in the presence of 10 μM or 100 μM odorant (light and dark gray colors, respectively). The error bars show the standard deviation and were calculated from three independent experiments. Statistical analysis was performed using a bilateral Student’s t-test. Asterisks indicate statistical significance (*P < 0.05 and **P < 0.01). The best inhibitors are indicated by the highest inhibition rates.

To detect the different GST functions (glutathione conjugation, isomerization and ligandin), we performed an assay based on the inhibition by an odorant molecule of the CDNB glutathione conjugation activity by GST. GST activity was measured using a chromophoric molecule. This assay was previously successfully used to screen tasting molecules toward a Drosophila GST [22,31]. This assay advantageously allows the detection of any of the previously described interactions of odorant molecules, such as substrate or non-substrate (ligandin function). Twenty different odorant molecules were tested belonging to different chemical classes. This odorant list includes odorants previously tested on a rat OE extract [15] and new odorants including stereoisomers. The rat GSTM2 activity test was performed in the presence of 1 mM CDNB and GSH with the addition of 10 μM or 100 μM odorant (Fig 8). Our results indicate that various odorant molecules inhibited GSTM2 activity (CDNB conjugation) (Fig 8). The odorants with the highest inhibition rates, including trans-2-nonenal, S-carvone, cinnamaldehyde and limonene oxide. At 100 μM, additional odorants able to interact with GSTM2 were identified as trans-2-hexen-1-al, R-carvone, citral and beta-ionone. The level of inhibition of GSTM2 activity was related to the odorant concentration: as expected, the inhibition activity was stronger at 100 μM odorant concentration compared to 10 μM odorant concentration. For example, S-carvone inhibited 26% of CDNB activity at 10 μM and 84% at 100 μM. The strong inhibition of S-carvone at 100 μM indicates that the S-carvone affinity is probably better than the CDNB_,_ especially since CDNB was used at a 10-fold higher concentration (1 mM) in this assay. Interestingly, S-carvone strongly inhibited GSTM2 compared to R-carvone, indicating that the enzyme is odorant stereospecific. This stereospecificity was confirmed for limonene oxide; indeed, when the two stereoisomers were independently tested, (+) limonene oxide appeared to be a better inhibitor compared to (-) limonene oxide (Fig 8). With 64% inhibition at 10 μM, (+) limonene oxide appears to be the best odorant inhibitor among the tested odorants in this study.

### Measure of the rat GSTM2 enzymatic activity toward odorants using high-performance liquid chromatography

**Fig 9:**
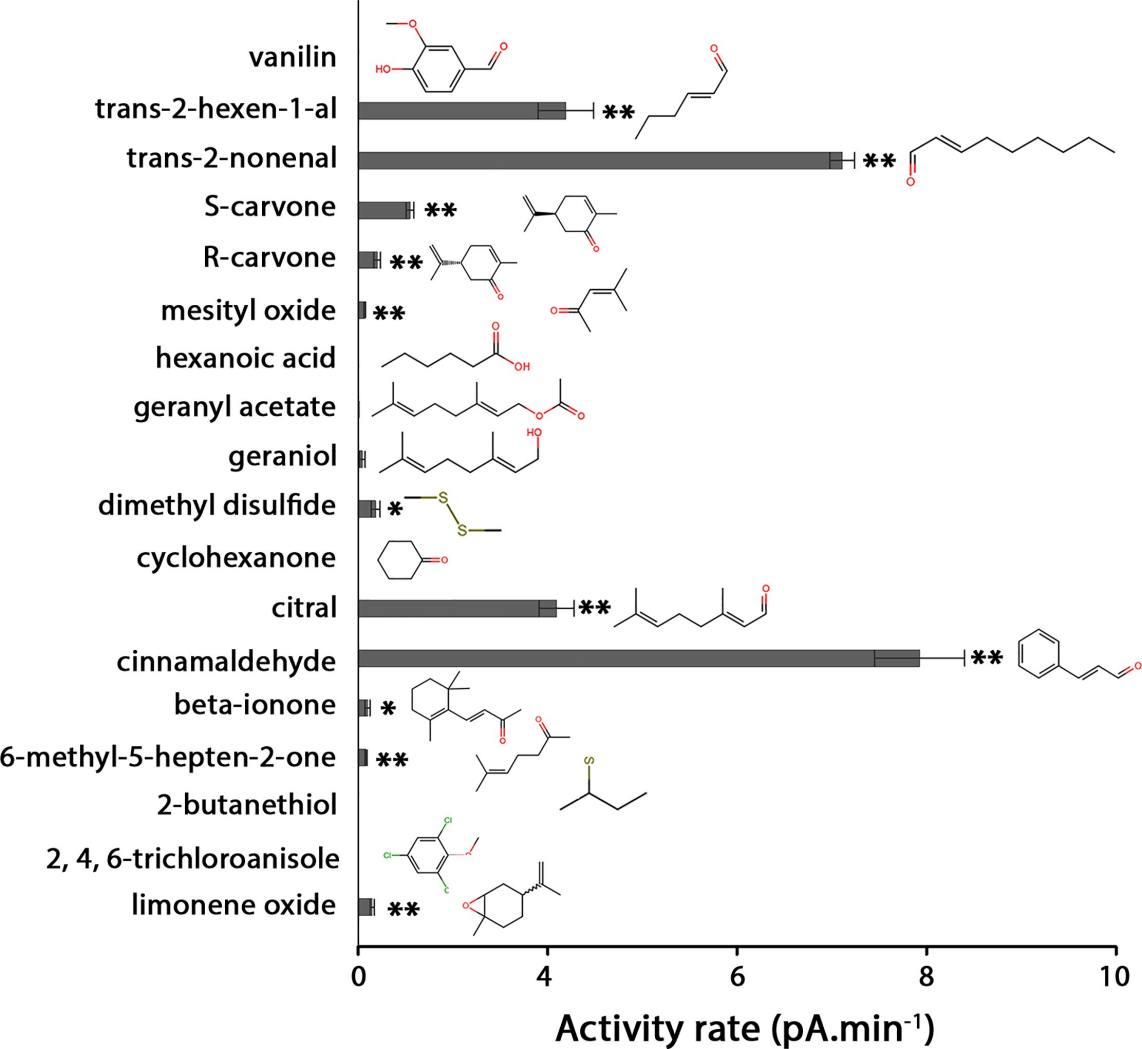
Formation of the odorant glutathione conjugation. Enzymatic glutathione conjugation was measured using high-performance liquid chromatography in the presence of different odorants, glutathione and the recombinant rat GSTM2. To determine the enzymatic glutathione conjugation, the non-enzymatic conjugation was subtracted. The error bars show the standard deviation and were calculated from three independent experiments. Statistical analysis was performed using a bilateral Student’s t-test. Asterisks indicate statistical significance (*P < 0.05 and **P < 0.01).

The glutathione conjugation of an odorant leads to the production of a new and more hydrophilic metabolite. An enzyme by definition catalyzes a chemical reaction already occurring spontaneously. *In vitro* conditions used to obtain a quantifiable product of the enzymatic reaction favor the spontaneous chemical reaction; consequently, for each experiment, we also measured the spontaneous non-enzymatic product formation. This non-enzymatic product formation is subtracted from the product in the presence of GSTM2, enabling us to observe the reaction resulting only from the enzymatic activity (Fig 9). For cyclohexanone, geraniol, vanillin or 2,4,6-trichloroanisole, no significant glutathione conjugation, either spontaneous or enzymatic, was observed (Table 3). For 2-butanethiol, geranyl acetate and hexanoic acid, a chemical reaction occurred, but neither enzymatic reaction was measured (Table 3). Additionally, any of these seven molecules inhibited recombinant rat GSTM2 (Fig 8). Conversely, for the 11 other tested molecules, the enzymatic reaction was higher compared to the spontaneous reaction. All 11 molecules showing enzymatic activity are also as expected inhibitors of CDNB activity. The technique is not accurate for comparing the enzymatic activity molecule-to-molecule. However, for the same molecule, it is possible to compare the spontaneous chemical reaction with the enzymatic reaction. Then, citral, limonene oxide, S-carvone and trans-2-nonenal are highly conjugated in the presence of the enzyme. These four molecules are also among the best inhibitors of CDNB activity.

**Table 3:** Enzymatic and spontaneous glutathione conjugation of odorants.

Molecule	Enzymatic activity (pA.min^-1^)	Spontaneous chemical conjugation (pA.min^-1^)
limonene oxide	0.15 +/- 0.02	0.02 +/- 0.01
2,4,6-trichloroanisole	0.00 +/- 0.00	0.00 +/- 0.00
2-butanethiol	0.00 +/- 0.00	2.80 +/- 0.60
6-methyl-5-hepten-2-one	0.08 +/- 0.01	0.02 +/- 0.01
beta-ionone	0.10 +/- 0.03	0.08 +/- 0.01
cinnamaldehyde	5.93 +/- 0.47	4.70 +/- 0.18
citral	2.09 +/- 0.18	0.33 +/- 0.11
cyclohexanone	0.00 +/- 0.00	0.00 +/- 0.00
dimethyl disulfide	0.18 +/- 0.05	2.96 +/- 0.05
geraniol	0.04 +/- 0.03	0.00 +/- 0.00
geranyl acetate	0.00 +/- 0.00	0.03 +/- 0.01
hexanoic acid	0.00 +/- 0.00	0.02 +/- 0.01
mesityl oxide	0.07 +/- 0.01	0.16 +/- 0.02
R-carvone	0.20 +/- 0.04	0.04 +/- 0.01
S-carvone	0.55 +/- 0.04	0.03 +/- 0.01
trans-2-nonenal	5.11 +/- 0.13	0.50 +/- 0.20
trans-2-hexen-1-al	2.19 +/- 0.29	2.54 +/- 0.12
vanillin	0.00 +/- 0.00	0.00 +/- 0.00

Enzymatic glutathione conjugation was measured in the presence of odorants, glutathione and the recombinant rat GSTM2. To determine the enzymatic glutathione conjugation, the non-enzymatic conjugation (chemical conjugation) was subtracted from the total. The standard deviation was calculated from three independent experiments.

## Discussion

Because of the constant exposure of the OE to various airborne molecules, this tissue, which is also a known pathway to the brain, requires protection. Chemical protection involves the elimination of exogenous molecules combined with the regulation of the oxidative stress promoted by certain molecules. The proteomic analysis conducted on the rat nasal mucus revealed the presence of numerous major antioxidant enzymes such as peroxiredoxin, glutathione peroxidase, catalase and superoxide dismutase. Additionally, the protective role of the mucus required enzymes involved in the metabolization of the most reactive molecules. The presence of a large number of enzymes involved in aldehyde oxidation, such as aldehyde dehydrogenases, is in agreement with the high reactivity of aldehydes that require quick elimination to avoid damage. Reduced glutathione plays a role as an essential cofactor for both the antioxidant and metabolizing enzymes [32]. As shown in this study, reduced glutathione can also spontaneously react with various odorants, including some aldehydes, such as cinnamaldehyde or trans-2-hexen-1-al. In contrast, glutathione does not react with all aldehydes, such as vanillin. The heterogeneous expression of GST in tissue is generally linked to specific requirements in this tissue. However, in mammals, including rodents, the GST classes are selectively expressed in tissues [33]. In the human OE, GSTP is highly expressed followed by GSTA; mu class GSTs are not detected in the human OE [34,35]. These first observations were also supported by the mass spectrometry analysis of human olfactory mucus, since only GSTA1 and GSTP1 were detected in this fluid [1]. A second recent proteomic analysis of human mucus confirmed this observation and additionally showed better expression of GSTP1 in the mucus of the youngest subjects [36]. In young rabbits, GSTA1, GSTM1 and GSTP1 were detected [37]. In the rat OE, a partial purification reveals that two GSTs of the mu class are major enzymes [15]. The proteomic analysis presented in this study supports this observation; indeed, GSTM1 and GSTM2 were detected with a large number of peptides. Moreover, the immunohistochemistry analysis of the OE tissue shows GSTMs expression in OE and in the olfactory mucus. Western blot analysis failed to detect GSTMs in the mucus using the same antibodies, possibly due to the lower sensitivity of this technique compared to immunohistochemistry. The antibodies specific to GSTMs revealed their expression in different cell types, including sustentacular or basal cells, as well as the duct of Bowman’s glands involved in olfactory mucus secretion. GSTMs expression is specific to the OE compared to the respiratory epithelium, supporting a functional role in this particular tissue. Western blot analysis clearly revealed the presence of GSTMs in the OE but a limited amount of GSTAs, and immunohistochemistry revealed low staining for GSTAs in the OE. However, even if the antibodies used to detect the GSTAs are efficient in detecting recombinant human GSTA1, it cannot be excluded that the low detection is due to a low affinity for the rat GSTAs. Additionally, although the mass spectrometry analysis was not quantitative, the GSTAs were detected in only one of the three rats. A previous study showed similar intense staining of rat OE using antibodies against GSTMs and GSTAs [25]. These polyclonal antibodies were obtained using purified GST directly from human liver due to the limited access to recombinant protein at this time, increasing the risk of cross contamination, which can also explain the difference in staining compared to this study. Our study pinpoints interspecies GST profile differences suggesting interspecies differences in chemoprotection and chemoperception.

The odorant metabolization by GSTMs localized in the OE or the mucus contributes to decreasing their availability and changing their ability to activate the olfactory receptors. Due to their structure, the resulting glutathione-conjugated metabolites are unlikely to activate new or the same olfactory receptors. Thus, we hypothesize that the protective role of GSTMs in this tissue also contributes to the termination of the olfactory signal. GSTM2 metabolizes a large variety of odorants that are structurally different. Odorants can probably be metabolized more or less efficiently. Moreover, assays based on the inhibition of CDNB conjugation revealed that some enzymatically conjugated odorants can also be inhibitors such as S-carvone or limonene oxide. Further studies will allow the determination of the kinetic parameter of this enzyme for these odorants. Interestingly, it has been previously shown that molecules can simultaneously bind the active site and ligandin site [38]; in this context, it cannot be excluded that an odorant acting as a substrate also binds the L-site. All of the tested odorants in this study displaying enzymatically catalyzed glutathione conjugation also inhibited CDNB activity, suggesting that all of them are at least substrate. The binding of a molecule in the ligandin site can drastically reduce GST activity [39]. Additionally, future studies should not exclude odorants that are not substrates but only inhibitors of GST activity. In a physiological context, odorants are in mixture, and the catalysis of each odorant depends on its enzymatic parameters for a given GST but also depends on the kinetic parameters of the other odorants for the same enzyme. The impact of such a mechanism on olfactory perception remains to be elucidated.

In conclusion, our work highlights (i) the presence of xenobiotic metabolizing enzymes and GSTs, particularly in the near receptor environment, including epithelium cells and mucus, (ii) the major expression of mu class GSTs in olfactory tissues and mucus, and (iii) the function of GSTs in the modulation of odorant availability, taking in charged odorants in a metabolizing process.

## Supporting information

S1 Checklist Reporting of *in vivo* experiments. (PDF)

S1 Appendix Alignment of the synthetic and natural DNA sequences coding for rat GSTM2. The *NdeI* and *SacI* restriction sites are highlighted.
(DOCX)

S1 Table Identification of rat nasal mucus enzymes. For each rat, the total number of peptide, the percentage of sequence coverage, the peptide sequence, the total number of spectra and the protein function are indicated.
(XLSX)
