# Supplementary material for: Characterization of rat glutathione transferases in olfactory epithelium and mucus
Source: PLoS One. 2019 Jul 24;14(7):e0220259. doi: 10.1371/journal.pone.0220259 (PMC6656353; doi:10.1371/journal.pone.0220259)
Supplement: S1 Appendix — The NdeI and SacI restriction sites are highlighted. (DOCX) [file pone.0220259.s002.docx]

10 20 30 40 50 60 70

....|....|....|....|....|....|....|....|....|....|....|....|....|....|

**Synthetic sequence**  **CATATGCCGATGACCCTGGGTTACTGGGACATCCGTGGTCTGGCGCACGCGATCCGTCTGTTCCTGGAAT**

**GSTM2 RNA (NM_177426.1)** **---ATGCCTATGACACTGGGTTACTGGGACATCCGTGGGCTGGCTCACGCCATTCGCCTGTTCCTGGAGT**

80 90 100 110 120 130 140

....|....|....|....|....|....|....|....|....|....|....|....|....|....|

**Synthetic sequence**  **ACACCGATACCAGCTACGAAGATAAAAAATACAGCATGGGTGATGCGCCGGATTACGATCGTAGCCAGTG**

**GSTM2 RNA (NM_177426.1)** **ATACAGACACAAGCTATGAGGACAAGAAGTACAGCATGGGGGATGCTCCCGACTATGACAGAAGCCAGTG**

150 160 170 180 190 200 210

....|....|....|....|....|....|....|....|....|....|....|....|....|....|

**Synthetic sequence**  **GCTGAGCGAAAAATTCAAACTGGGTCTGGATTTCCCGAACCTGCCGTACCTGATCGATGGTAGCCACAAA**

**GSTM2 RNA (NM_177426.1)** **GCTGAGTGAGAAGTTCAAACTGGGCCTGGACTTCCCCAATCTGCCCTACTTAATTGATGGGTCACACAAG**

220 230 240 250 260 270 280

....|....|....|....|....|....|....|....|....|....|....|....|....|....|

**Synthetic sequence**  **ATCACCCAGTCTAACGCGATCCTGCGTTACCTGGGCCGTAAACACAACCTGTGCGGTGAAACCGAAGAAG**

**GSTM2 RNA (NM_177426.1)** **ATCACCCAGAGCAATGCCATCCTGCGCTACCTTGGCCGGAAGCACAACCTTTGTGGGGAGACAGAGGAGG**

290 300 310 320 330 340 350

....|....|....|....|....|....|....|....|....|....|....|....|....|....|

**Synthetic sequence**  **AACGTATCCGTGTTGATGTTCTGGAAAACCAGGCGATGGATACCCGTCTGCAGCTGGCGATGGTTTGCTA**

**GSTM2 RNA (NM_177426.1)** **AGAGGATTCGTGTGGACGTTTTGGAGAACCAGGCTATGGACACCCGCCTACAGTTGGCCATGGTCTGCTA**

360 370 380 390 400 410 420

....|....|....|....|....|....|....|....|....|....|....|....|....|....|

**Synthetic sequence**  **CAGCCCGGATTTCGAACGTAAAAAACCGGAATACCTGGAAGGCCTGCCGGAAAAAATGAAACTGTACTCT**

**GSTM2 RNA (NM_177426.1)** **CAGCCCTGACTTTGAGAGAAAGAAGCCAGAGTACTTAGAGGGTCTCCCTGAGAAGATGAAGCTTTACTCC**

430 440 450 460 470 480 490

....|....|....|....|....|....|....|....|....|....|....|....|....|....|

**Synthetic sequence**  **GAATTCCTGGGTAAACAGCCGTGGTTCGCGGGCAACAAAATCACCTACGTCGATTTTCTGGTTTACGATG**

**GSTM2 RNA (NM_177426.1)** **GAATTCCTGGGCAAGCAGCCATGGTTTGCAGGGAACAAGATTACGTATGTGGATTTTCTTGTTTACGATG**

500 510 520 530 540 550 560

....|....|....|....|....|....|....|....|....|....|....|....|....|....|

**Synthetic sequence**  **TGCTGGACCAGCACCGTATTTTCGAACCGAAATGCCTGGATGCCTTCCCGAATCTGAAAGACTTCGTTGC**

**GSTM2 RNA (NM_177426.1)** **TCCTTGATCAACACCGTATATTTGAACCCAAGTGCCTGGACGCCTTCCCAAACCTGAAGGACTTCGTGGC**

570 580 590 600 610 620 630

....|....|....|....|....|....|....|....|....|....|....|....|....|....|

**Synthetic sequence**  **GCGTTTTGAAGGCCTCAAGAAAATCTCTGACTACATGAAATCCGGTCGTTTCCTGAGCAAACCGATCTTC**

**GSTM2 RNA (NM_177426.1)** **TCGGTTTGAGGGCCTGAAGAAGATATCTGACTACATGAAGAGCGGCCGCTTCCTCTCCAAGCCAATCTTT**

640 650 660

....|....|....|....|....|....|....|....

**Synthetic sequence**  **GCGAAAATGGCGTTCTGGAACCCGAAGTAATAAGAGCTC**

**GSTM2 RNA (NM_177426.1)** **GCAAAGATGGCCTTTTGGAACCCAAAGTAG---------**
